# Reduction of Precautionary Behaviour following Vaccination against COVID-19: A Test on a British Cohort

**DOI:** 10.3390/vaccines10060936

**Published:** 2022-06-12

**Authors:** Olivier Desrichard, Lisa Moussaoui, Nana Ofosu

**Affiliations:** Faculty of Psychology and Educational Sciences, University of Geneva, 1205 Geneva, Switzerland; lisa.moussaoui@unige.ch (L.M.); nana.ofosu@unige.ch (N.O.)

**Keywords:** risk homeostasis, risk compensation, precautionary behaviour, COVID-19, vaccination

## Abstract

Background: There is a risk that people vaccinated against COVID-19 will drop or reduce their precautionary behaviours (i.e., a phenomenon of risk homeostasis). Our aim is to assess the occurrence of this effect in a cohort of UK participants who were interviewed 141 days before and 161 days after the start of the vaccination programme. Methods: Of the 765 people who could be followed up before and after the start of the programme and whose vaccination status was known, 178 had not received any injection and 583 were more or less advanced in the process (one vs. two doses since less vs. more than 14 days). The frequency of 14 precautionary behaviours was assessed at both times of measurement, as well as potential covariates (gender, age, comorbidities and history of COVID-19). Results: Controlling for covariates, we didn’t find more decrease in precautionary behaviours among vaccinated individuals, regardless of how far along they were in the process. Conclusion: The results observed in this sample show little risk for a massive change in behaviours among early vaccinated individuals. The pressure to adopt precautionary behaviours remains strong and probably prevents the emergence of a risk homeostasis effect.

## 1. Introduction

Despite the hope of vaccination being a preventive solution in the fight against the Coronavirus disease 2019 (COVID-19) pandemic, the World Health Organization (WHO) recommends that barrier measures not be abandoned, as vaccinated individuals could still become infected (but avoid a severe form of the disease) or continue to transmit severe acute respiratory syndrome coronavirus 2 (SARS-CoV-2) and its variants [1]. It would therefore be a threat to public health if vaccinated people reduced or even abandoned precautionary behaviours, such as wearing masks or washing their hands. Different labels have been used (risk compensation, risk homeostasis, the Peltzman effect) to express the idea that individuals will reduce their precautionary behaviour when the intrinsic risk of the situation decreases [2]. As the goal of this adjustment is not to reduce the risk faced by the individual to zero, but rather to conserve it at an acceptable level (called the target risk) that allows for a trade-off between the cost of precautions and the benefits of risk-taking, the term “risk homeostasis” seems to be preferable. Being vaccinated may represent a new element that may upset the balance that had been established over the initial months of the pandemic in the adoption of precautionary behaviours, and lead people to reconsider the necessity of these actions. Scientific voices have discussed this negative side of vaccination [3,4,5,6]. Rubin et al. cite epidemiological data from two studies showing that the probability of testing positive to SARS-CoV-2 increased after the first dose of vaccination [7,8]. Studies on other vaccines (influenza and Lyme disease) also suggest a possible rebound in risk-taking after receiving the injection [9,10]. On the other hand, observing a reduction in precautionary behaviours when the adoption of new safety measures makes the environment safer is far from a universal phenomenon. The conclusion of the reviews in various fields rather shows that this effect is only occasionally observed [2,3,6]. For example, a review including 20 studies on human papillomavirus infection (HPV) vaccination and sexually transmitted infection (STI) rates showed no such effect [11].

Based on the discordant results in the literature, there is a need for more studies that examine the presence of a risk homeostasis effect in individuals vaccinated against COVID-19. To the best of our knowledge at the time of writing, only one study looked at whether people vaccinated against COVID-19 reduced their protective behaviour [12]. Results suggest that individuals do not substantially decrease compliance following vaccination. However, the authors acknowledge that results should be replicated on a longer follow-up period, with general population data not gathered during a period of strict lockdown.

In this study, we use longitudinal data collected over a period of 1 year to test this question, comparing the change in frequencies of 14 precautionary behaviours between March 2020 and June 2021, depending on the vaccination status of the respondent. In addition, we analysed potential changes in behaviour as a function of the number of doses received (1 or 2) and the time elapsed since vaccination (less or more than 14 days). Most COVID-19 vaccines require two injections separated by at least 14 days. After this period, the intrinsic risk decreases and should therefore be manifested by a decrease in precautionary behaviours in individuals who have received an injection (the first or second) for more than 14 days. Since the second injection leads to the lowest level of intrinsic risk, the decrease should be even greater among this group. 

## 2. Materials and Methods

All methods were carried out in accordance with relevant guidelines and regulations. The protocol of this study has been approved by the University of Geneva’s Committee for Ethical Research. The data are anonymous, and all participants signed an informed consent form.

### 2.1. Study Design, Setting, and Participants

The survey administered for this study is part of a larger research project assessing adherence to protective measures in a nationally representative UK sample of adults (initial N = 1006), for which data were regularly collected between spring 2020 and spring 2021, including the present study [13]. The sample was provided by Prolific, a platform for recruiting participants to take part in online studies [14]. All subjects were 18 years of age or older. There were no other inclusion criteria. Participants were remunerated GBP 1 for their participation in each wave. The initial survey was approved by the University of Geneva’s Ethical Review Board and was registered on AsPredicted [15] prior to its launch. 

For the present study, a new wave was administered in 2021 between 28 May and 15 June, eleven days after the UK began easing lockdown restrictions and 161 days after the start of the vaccination programme. The full survey is available at OSF [16]. In order to control for past adherence to protective measures, and for age and sex, we included data from the previous wave, which took place in July 2020, 141 days before the start of the vaccination programme.

Nine hundred and thirty-one participants who had taken part in the prior wave (July 2020, hereafter referred to as T1) of the research project, and who had agreed to be contacted for further surveys, were invited to complete the new survey (May 2021, hereafter referred to as T2) entitled “Wave 4—Perception of COVID-19 and Protective Behaviours”. The response rate of eligible participants was 82.2% (N = 765). Information on the sample is available in Table 1.

### 2.2. Procedure

Eligible participants received a message inviting them to complete the survey. Those who accepted began by completing the informed consent form. Following this, participants answered questions regarding COVID-19. At the end of the survey, participants had the opportunity to indicate whether they had responded seriously (with no consequences for their remuneration; “Yes” = 100%), and to leave a comment if they so wished.

### 2.3. Measures

Descriptive statistics that are not in the text are presented in Table 1. We use T1 and T2 to refer to the observations made in T1 (20/07/22) and T2 (21/05/28) of the study, respectively.

*Vaccination status (T2).* Participants were asked whether they had received at least one dose of the COVID-19 vaccine. Those who responded that they were vaccinated indicated how many doses they had received (two doses; one dose and will not get a second one because one dose is enough to be protected; one dose and waiting for the second dose). People with one dose with no expectation of a second dose (N = 5, 0.9%) were pooled with people with two doses.

*Days since last dose (T2).* Participants who had received at least one dose of the vaccine indicated how many days had elapsed since their last dose (M = 30.7, SD =23.7).

*Type of vaccine (T2).* Participants reported which vaccine they had received (Oxford-AstraZeneca: 66.2%; Pfizer-BioNTech: 31.8%; Moderna: 1.5%; I don’t know/remember: 0.5%).

*Comorbidities (T2).* Participants noted whether they were at risk for a severe form of COVID-19 due to comorbidity.

*COVID-19 status (T2).* One item recorded whether participants believed they had had COVID-19. The response options were as follows: “I really think I haven’t had it”, “I probably have not had it”, “I may have had it but I’m unsure”, “I really think I had it”, “Yes, I had a medical diagnosis or a test that confirmed it”, or “Not applicable”. Responses were recoded as 1 = I really think I haven’t had it; 2 = I probably have not had it; 3 = I may have had it but I’m unsure; 4 = Yes, I had a medical diagnosis or a test that confirmed it or I really think I had it.

*Perceived protection (T2).* One item recorded the extent to which the person felt protected from COVID-19 given their current vaccination status. For example, unvaccinated individuals rated the item “With your current medical status (0 dose), you consider yourself protected from COVID-19 or its severe form at...”. The vaccination status was replaced by one or two doses for participants who had already received one or two injections, respectively, regardless of when. The response options were as follows: 0% (not at all protected), 10%, 20%, 30%, 40%, 50%, 60%, 70%, 80%, 90%, or 100% (completely protected). 

*Protective measures (T1 and T2).* Fourteen different behaviours were assessed. These had initially been based on the UK’s NHS recommendations at the time of the first survey launched in 2020. From a list of 14 behaviours (see list in Table 2), participants indicated the extent to which they had behaved in that way, on a typical day or on average, during the last ten days. Responses were given on a 5-point Likert scale ranging from 1 (“Never”) to 5 (“Very often” or “Always”, depending on the item). An additional response option was included (“Don’t know/Not applicable”) and treated as missing data.

### 2.4. Outcomes

The primary outcome of our study was the change in the 14 precautionary behaviours before (T1) and after (T2) the start of the vaccination programme, depending on the participant’s vaccination status (number of doses) at T2.

### 2.5. Statistical Analysis

We performed a series of 14 repeated-measures ANOVAs with the time of measurement (T1 = before vs. T2 = after the start of the vaccination programme) as the repeated measures factor. The number of doses at T2 was entered as a moderator of change between T1 and T2. Participants’ age, sex, comorbidities, and COVID-19 exposition were entered as covariates. We used Bonferroni corrections to set the *p*-value at which a difference was considered statistically significant. We used a *p*-value of 0.003 for tests of the 14 interaction effects between measurement time and number of doses. We used a *p*-value of 0.01 to test the 5 simple effects within each analysis.

## 3. Results

Of the 931 participants in T1, 765 (82.2%) participated in T2, of which 761 had identified vaccination status. A priori power analysis performed with G * power [17] indicates there is an 81% chance of correctly rejecting the null hypothesis of no significant effect of the interaction (number of doses = 5 * time of measurement = 2), with 31 participants per group, for a total of 155 participants. With a total of 765 participants and a minimum of 48 participants per group, our sample is adequately powered to detect a small effect size (η^2^*p* = 0.02). There was no difference in sex ratio between participants and non-participants in T2 (chi-square = 1.63, df = 2, *p* = 0.444). Non-participants in T2 were younger (m = 39.9) than participants (m = 48.1, t = −6.55, df = 919, *p* < 0.001). Table 1 shows the distribution of participants on measures according to vaccination status. The mean age and sex ratio in the present sample are similar to data from the 2018 census of the UK Office for national statistics (m-age = 45.79, SD = 15.52, 51.15% women).

**Figure 1:**
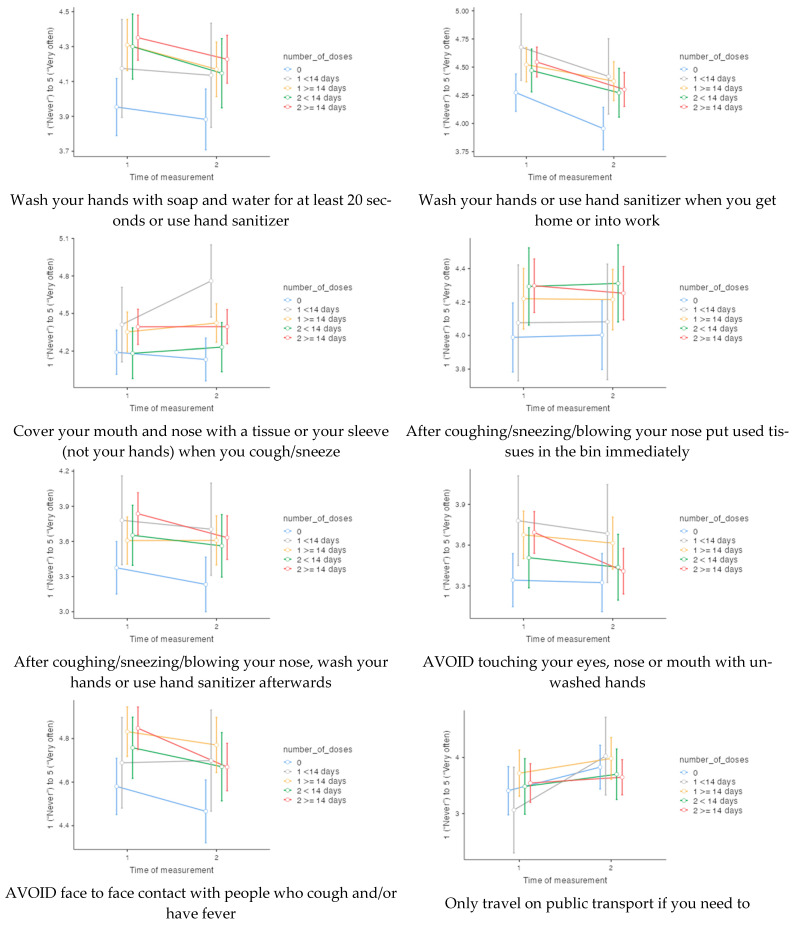

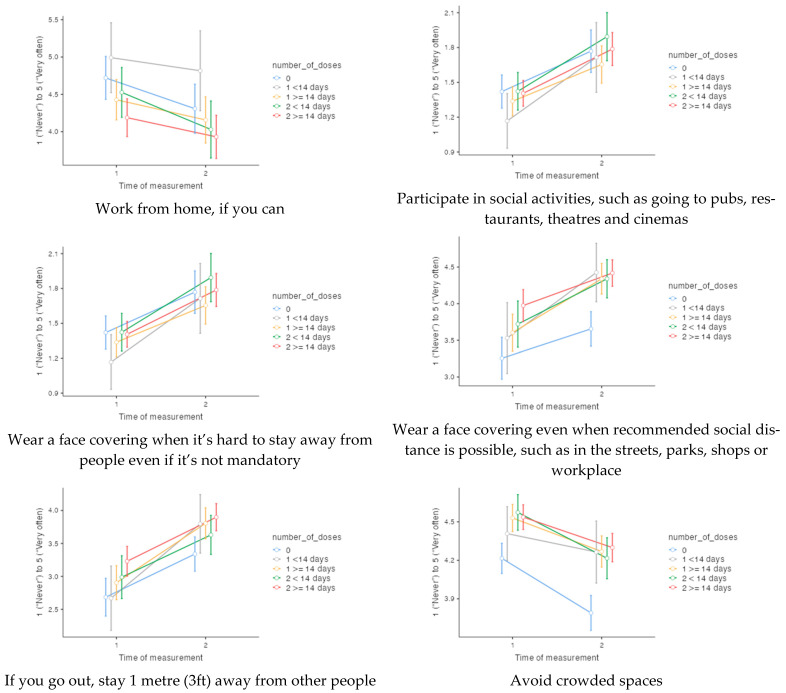
Frequencies of 14 precautionary behaviours at time of measurement 1 (141 days before the start of the vaccination programme) and time of measurement 2 (161 days after the start of the vaccination programme), according to number of doses at time of measurement 2.

Figure 1 shows the estimated means at T1 and T2 according to vaccination status. Table 1 provides a summary of the results of the statistical analysis. After controlling for covariates, we observe no significant change in the adoption of precautionary behaviours between T1 and T2, with the exception of “Avoid crowded spaces”, which significantly decreases. We also do not observe an effect of vaccination status. However, the analysis of simple effects (see Table 2) reveals some differences. In particular, the group of participants with full vaccination coverage (two doses for more than 14 days) showed nine significant changes between T1 and T2, against five in the group of non-vaccinated participants (zero dose) and three in the group of participants at the beginning of vaccination (1 dose for less than 14 days). Note that two of the changes between T1 and T2, both concerning the wearing of masks, are in the direction of an increase in precautionary behaviour.

## 4. Discussion

From the point of view of the risk homeostasis literature, there is a legitimate concern that vaccinated people will reduce or abandon their precautionary behaviour with regards to COVID-19. This would represent a real threat to the control of this epidemic and would justify investing in communication effort. However, there are few data to justify public health actions to address this problem. In the present study, we investigated the change in people’s behaviour at different stages of their COVID-19 vaccination (including not being vaccinated), between before (T1) and after (T2) the start of the vaccination programme in Britain. Despite an increase in the feeling of protection as participants progress through the vaccination process, our results do not document a risk homeostasis effect. Although there are some changes between T1 and T2 (both in the direction of a decrease in some precautionary behaviours and an increase in others), there were no significant interactions between change and vaccinal status. Local group analyses (simple effects) suggest that the fully vaccinated group would reduce their precautionary behaviour more than the other groups, particularly for some hand-washing behaviours and for the behaviour “AVOID face to face contact with people who cough and/or have fever”. However, the effect sizes representing the change between T1 and T2 are small, indicating that, at worst, the magnitude of the problem is negligeable from a practical standpoint.

Our results replicate those of Wright et al. [12] and also provide original insights. We show that the lack of effect they observed was not due to having measured precautionary behaviours during a period of strict confinement. In addition, we used a more specific and comprehensive measure of precautionary behaviour and a more detailed distinction between the different vaccination statuses. We also show that, while the feeling of protection is impacted by vaccination status, it has no effect on protective behaviour.

As the literature indicates, due to situation-specific factors, the risk homeostasis effect does not always occur. In the case of COVID-19, one explanation may lie in the fact that protecting oneself and others is still a widespread leitmotif in the population and in authorities’ communication. Thus, social pressure may be exerted and reduce the motivation or sense of control of the vaccinated to decrease their precautionary behaviour, two factors that modulate the occurrence of a risk homeostasis effect [2]. An additional explanation could be that even though the intrinsic risk is decreasing, the perceived risk remains high enough for vaccinated people to continue protecting themselves. Nevertheless, our data show that fully vaccinated people consider themselves to be very protected (m = 79.4%), without massively abandoning their protective behaviours. A secondary result may also shed light on the reasons for the absence of a risk homeostasis effect. Indeed, it appears that for many of the precautionary behaviours, the people who were not vaccinated at T2 were also those who were already the least precautionary at T1, regardless of sex, age, comorbidity, and history of COVID-19. It is likely that those who were vaccinated first are also those who are most motivated to protect themselves, which could explain their continued precautionary behaviour even after vaccination. Future studies will be needed to verify that late-vaccinated individuals are not more susceptible to a risk homeostasis effect.

A limitation of our study is the use of a self-reported measure of the precautionary behaviours. Although this method is widely used, it can only provide an approximate measure of actual behaviour. However, meta-analyses in other areas (e.g., sedentary behaviours [18], medical adherence [19], and PAP and mammography screening [20]) indicate that objective and self-report measures of behaviours have good convergent validity.

We can also consider that government health policy during this period (e.g., lockdown, communication, sanctions) may have largely influenced precautionary behaviour. Seasonality is also a factor that may have interfered with changes in behaviour. These elements limit the conclusions that can be made about the changes between T1 and T2, which may reflect the effect of environmental constraints as much as the motivation of participants to change their precautionary behaviour. However, there is no reason to believe that these external factors interacted with the risk homeostasis effect. All things being equal, and in a period when restrictions are easing (which is the case for our participants in T2), one would have expected precautionary behaviour to change more among vaccinated participants. Our results do not support this conclusion.

## 5. Conclusions

From a behavioural science perspective, it is legitimate to carefully track the impact that a solution such as vaccination may have on the abandonment of precautionary behaviours. The current data that we assess in this article indicate that the spread of vaccination does not threaten the effectiveness of the behavioural recommendations used in the fight against the COVID-19 epidemic. However, this is probably the result of continued communication about the seriousness of the pandemic and the importance of barrier measures. It is therefore strongly recommended that the authorities themselves do not relax their efforts on protective behaviour or communicate too quickly on the effectiveness of vaccination as a solution to the crisis.

## Figures and Tables

**Table 1:** Description of the cohort.

		Vaccination Status (Row Percent)				
	All	0 Dose	1 Dose < 14 Days	1 Dose ≥ 14 Days	2 Doses < 14 Days	2 Doses ≥ 14 Days
N (%) *	765 (100%)	178 (23.4)	48 (6.5)	176 (23.1)	108 (23.1)	251 (33)
Age years						
Mean (SD)	48.1 (14.6)	36 (13.4)	35 (6.9)	48 (9.93)	54.5 (9.23)	56.4 (13.4)
Sex						
Male (%)	353 (47.6)	85 (24.4)	27 (7.8)	86 (24.7)	46 (13.2)	104 (29.9)
Female (%)	388 (52.4)	84 (21.8)	19 (4.9)	85 (22.1)	57 (14.8)	140 (36.4)
Comorbidities						
Yes (%)	137 (17.9)	16 (11.7)	1 (0.7)	28 (20.4)	18 (13.1)	74 (54)
No (%)	628 (82.1)	162 (26)	47 (7.5)	147 (23.6)	90 (14.4)	177 (28.4)
COVID-19 Status **						
1 (%)	461 (61.1)	88 (19.2)	28 (6.1)	107 (23.4)	56 (12.2)	179 (39.1)
2 (%)	135 (17.9)	37 (27.8)	10 (7.5)	27 (20.3)	28 (21.1)	31 (23.3)
3 (%)	83 (11)	22 (26.5)	7 (8.4)	22 (26.5)	13 (15.7)	19 (22.9)
4 (%)	75 (9.9)	28 (37.3)	3 (4)	17 (22.7)	9 (12)	18 (24)
Perceived protection						
Mean (SD)		41.2 (34)	48.8 (21.9)	53.8 (17.3)	70.8 (16.4)	79.4 (14.8)
Educational Level						
1 (%)	10 (1.3)	3 (30)	0 (0)	2 (20)	2 (20)	3 (30)
2 (%)	100 (13.4)	13 (13.3)	2 (2)	31 (31.6)	16 (16.3)	36 (36.7)
3 (%)	126 (16.9)	50 (40)	9 (7.2)	20 (16.0)	17 (13.6)	29 (23.2)
4 (%)	99 (13.3)	16 (16.3)	0 (0)	24 (24.5)	14 (14.3)	44 (44.9)
5 (%)	269 (36.2)	65 (24.4)	18 (6.8)	58 (21.8)	38 (14.3)	87 (32.7)
6 (%)	119 (16.0)	22 (18.6)	14 (11.9)	30 (25.4)	15 (12.7)	37 (31.4)
7 (%)	21 (2.8)	2 (9.5)	3 (14.3)	6 (28.6)	2 (9.5)	8 (38.1)

N = total number of individuals; SD = Standard Deviation. * Variations in totals are because of missing data. ** 1 = I really think I haven’t had it; 2 = I probably have not had it; 3 = I may have had it but I’m unsure; 4 = Yes, I had a medical diagnosis or a test that confirmed it or I really think I had it.

**Table 2:** Summary of the statistical analysis.

			Simple Effects of Time of Measurement				
Outcomes	Main Effect of Time of Measurement	Time of Measurement * Number of Doses	0 Dose	1 < 14 Days	1 ≥ 14 Days	2 < 14 Days	2 ≥ 14 Days
Wash your hands with soap and water for at least 20 s or use hand sanitizer	*p* = 0.0012 η^2^*p* = 0.015 * (−)	*p* = 0.9322 η^2^*p* = 0.001	*p* = 0.3845	*p* = 0.7783	*p* = 0.0578	*p* = 0.1013	*p* = 0.0554
Wash your hands or use hand sanitizer when you get home or into work	*p* = 0.0001 η^2^*p* = 0.051 * (−)	*p* = 0.6112 η^2^*p* = 0.004	*p* = 0.0003 * (−)	*p* = 0.0992	*p* = 0.0718	*p* = 0.0520	*p* = 0.0006 * (−)
Cover your mouth and nose with a tissue or your sleeve (not your hands) when you cough/sneeze	*p* = 0.1947 η^2^*p* = 0.003	*p* = 0.1669 η^2^*p* = 0.010	*p* = 0.5389	*p* = 0.0274	*p* = 0.3996	*p* = 0.6531	*p* = 0.9867
After coughing/ sneezing/blowing your nose put used tissues in the bin immediately	*p* = 0.9541 η^2^*p* = 0.000	*p* = 0.9854 η^2^*p* = 0.001	*p* = 0.8888	*p* = 0.9718	*p* = 0.9537	*p* = 0.8696	*p* = 0.5620
After coughing/sneezing/ blowing your nose, wash your hands or use hand sanitizer afterwards	*p* = 0.0107 η^2^*p* = 0.010	*p* = 0.4376 η^2^*p* = 0.006	*p* = 0.1507	*p* = 0.6488	*p* = 0.9888	*p* = 0.4262	*p* = 0.0101 * (−)
AVOID touching your eyes, nose or mouth with unwashed hands	*p* = 0.0008 η^2^*p* = 0.016 * (−)	*p* = 0.1296 η^2^*p* = 0.010	*p* = 0.8462	*p* = 0.5520	*p* = 0.4733	*p* = 0.5128	*p* = 0.0001 * (−)
AVOID face to face contact with people who cough and/or have fever	*p* = 0.0351 η^2^*p* = 0.007	*p* = 0.6073 η^2^*p* = 0.004	*p* = 0.1674	*p* = 0.9398	*p* = 0.4004	*p* = 0.3326	*p* = 0.0046 * (−)
Only travel on public transport if you need to	*p* = 0.0026 η^2^*p* = 0.023 * (+)	*p* = 0.5008 η^2^*p* = 0.008	*p* = 0.0826	*p* = 0.0251	*p* = 0.2557	*p* = 0.4349	*p* = 0.5954
Work from home, if you can	*p* = 0.0005 η^2^*p* = 0.032 * (−)	*p* = 0.5656 η^2^*p* = 0.000	*p* = 0.0043 * (−)	*p* = 0.4535	*p* = 0.0457	*p* = 0.0030 * (−)	*p* = 0.0429
Participate in social activities, such as going to pubs, restaurants, theatres and cinemas	*p* < 0.0001 η^2^*p* = 0.128 * (+)	*p* = 0.4464 η^2^*p* = 0.006	*p* < 0.0001 * (+)	*p* = 0.0001 * (+)	*p* < 0.0001 * (+)	*p* < 0.0001 * (+)	*p* < 0.0001 * (+)
Wear a face covering when it’s hard to stay away from people even if it’s not mandatory	*p* < 0.0001 η^2^*p* = 0.143 * (+)	*p* = 0.0807 η^2^*p* = 0.013	*p* = 0.0043 * (+)	*p* = 0.0002 * (+)	*p* < 0.0001 * (+)	*p* < 0.0001 * (+)	*p* < 0.0001 * (+)
Wear a face covering even when recommended social distance is possible, such as in the streets, parks, shops or workplace	*p* < 0.0001 η^2^*p* = 0.168 * (+)	*p* = 0.2748 η^2^*p* = 0.008	*p* < 0.0001 * (+)	*p* < 0.0001 * (+)	*p* < 0.0001 * (+)	*p* = 0.0003 * (+)	*p* < 0.0001 * (+)
If you go out, stay 1 metre (3ft) away from other people	*p* < 0.0001 η^2^*p* = 0.087 * (+)	*p* = 0.1014 η^2^*p* = 0.011	*p* < 0.0001 * (+)	*p* = 0.1963	*p* < 0.0001 * (+)	*p* < 0.0001 * (+)	*p* < 0.0001 * (+)
Avoid crowded spaces	*p* ≤ 0.0001 η^2^*p* = 0.083 * (−)	*p* = 0.5227 η^2^*p* = 0.005	*p* = 0.0001 * (−)	*p* = 0.0821	*p* = 0.0044 * (−)	*p* = 0.0003 * (−)	*p* < 0.0001 * (−)

Note: * = significant after Bonferroni correction. The (+) and (−) indicate whether the change is towards an increase or decrease in behaviour.

## Data Availability

The dataset is available upon request.
